# Examining the relationship between empathetic leadership and organizational citizenship behavior in the architecture, engineering, and construction industry: the roles of altruistic attitude and caring ethical climate

**DOI:** 10.3389/fpsyg.2026.1767674

**Published:** 2026-02-24

**Authors:** Chenlin Liu, Siwei Sun, Guangya Ma, Qiang Ma

**Affiliations:** 1 Party School of China Rongtong Asset Management Group Co., Ltd., Beijing, China; 2 Department of Management and International Business, Business School, University of Auckland, Auckland, New Zealand; 3 School of Foreign Studies, Yiwu Industrial and Commercial College, Jinhua, China; 4 Chinese Academy of Environmental Planning, Beijing, China

**Keywords:** altruistic attitude, architecture, engineering, and construction industry, caring ethical climate, empathetic leadership, organizational citizenship behavior, real estate company

## Abstract

**Objective:**

Architecture, engineering, and construction industry plays an essential role in maintaining the sustainable development of the industry and the national economy. In recent years, companies have been forced to deal with challenges in performance and talent management due to the global recession. Although scholars have highlighted the potential role of empathy in management for its positive effect on healing people and enhancing organizational performance and sustainability, limited studies have been conducted in a leadership context in the architecture, engineering, and construction industry. Based on social information processing theory, we conduct a study in China to examine the mediating role of altruistic attitude and the moderating role of a caring ethical climate in the relationship between empathetic leadership and organizational citizenship behavior.

**Methods:**

Authors collect survey data from 296 employees in a Chinese real estate company to examine the moderated mediation model with regression analyses, supplemented by fuzzy-set qualitative comparative analysis.

**Findings:**

The results revealed that empathetic leadership was positively related to organizational citizenship behavior through altruism. Caring climate served as a significant moderator. Furthermore, the fuzzy-set qualitative comparative analysis reveals six configurational paths leading to high-level organizational citizenship behaviors and four configurational paths leading to low-level organizational citizenship behaviors.

**Conclusion:**

Based on survey data, this study can contribute to the understanding of empathetic leadership in architecture, engineering, and construction industry by verifying and extending the emotion-cognition process in the Chinese context. They showed that altruism facilitated the effectiveness of empathetic leadership and that caring climate could amplify the effect on employees’ organizational citizenship behavior. The results also contribute to organizational citizenship behavior literature by revealing the configurational effects of interactions among empathetic leader, team environment and individuals.

## 1 Introduction

Architecture, engineering, and construction (AEC) industry significantly affect the sustainable development of the industry and the national economy (Ni et al., 2022). The pressures from the appeal of environmental issues from government and non-government organizations, as well as the scarcity of land resources, make AEC companies face challenges in maintaining performance to survive in the acceleration of competition (Ni et al., 2022).

Literature has revealed that the economic challenges faced by AEC companies have overflowed to the employee level (Saleem et al., 2022) and brought huge pressure derived from supervisors’ control and fierce competition to employees. A report shows that in AEC companies, employees may face multiple challenges (Pucillo, 2023), such as uncontrollable external environments, long working hours, and communication stress with customers. These challenges significantly influence employees’ overall performance. These pressures make employees conduct non-compliant or opportunistic behaviors to avoid risks under uncertainty (Zhou et al., 2024; Liu et al., 2022; Li and Ning, 2022), exhibit unstable in-role behavior to avoid punishments due to individual decisions (Yan et al., 2025), and display low extra-role behavior as a result of loss avoidance (De Clercq and Belausteguigoitia, 2024). As the project nature of the AEC industry requires professionals to possess diverse expertise to deal with complex situations, this requirement necessitates collaboration during work and makes the organization’s operation highly dependent on coordination mechanisms beyond the scope of formal job responsibilities (Zhou and Wang, 2025). Moreover, technological advancements and intense competition have increased environmental uncertainty, making isolated, self-sufficient task execution insufficient to meet the complex project requirements in the AEC industry (Jacobsson et al., 2017). Different from in-role and unethical behaviors that can be regulated by formal systems and rules, extra-role behaviors cannot be forced by the organization (Organ, 1988). Given the urgency of dealing with complex environments and the collaborative nature of projects in the AEC industry, employees’ voluntary behaviors are essential to companies. This makes organizational citizenship behavior particularly important in the contemporary AEC environment and highlights the necessity of identifying how to prompt such autonomous behaviors.

In recent years, organizational management has increasingly emphasized the core status of people and paid attention to the role of emotion in the relationship between people. Past literature has verified that empathy, as a concept related to the establishment of interpersonal relationships in people’s lives, is closely linked with people’s basic needs and emotional feelings (Depow et al., 2021). A famous practitioner, Satya Nadella, who is the Microsoft CEO, states that empathy is a crucial source of organizational success because it can help people understand each other better and help each other satisfy basic and further needs to spark innovation and sustainability in organizations or even industries (Grant, 2018), which shows the feasibility and effectiveness of empathy in an organizational context. Past scholars call for more investigations of the outcomes of empathy in an organizational context (Clark et al., 2019).

In recent years, Kock et al. (2019) proposed the concept of empathetic leadership, referring to how much a leader understands a follower’s work situation, invests in emotional understanding, and provides emotional security for the follower. However, empirical research on empathetic leadership styles is still in its initial stages (Bani-Melhem et al., 2021). In the empathetic leadership context, scholars only found its effects on resilience (Wibowo and Paramita, 2022), turnover intention (Wibowo and Paramita, 2022) and job performance (Kock et al., 2019). Therefore, future investigations of empathetic leadership should be conducted, particularly in relation to a broader array of workplace outcomes. As organizational citizenship behavior is an essential predictor of the sustainability of organizational performance and the organizational climate and outcomes (Lee and Ha-Brookshire, 2018), this study focuses on the relationship between empathetic leadership and organizational citizenship behavior. This study regards individuals holding supervisory or managerial positions as ones exercising leadership in organizations and believes that those people’s practices can influence employees’ attitudes and actions.

As employees interact with others in their workplace daily, their perceptions are influenced by others’ reactions. This study draws on the social information processing theory (Salancik and Pfeffer, 1978) to investigate the influence of empathetic leadership and organizational citizenship behavior. Based on this theory, leaders are an essential source of information for employees, and social interaction helps employees deepen or form an understanding of their own needs, values, and perceptions, which will determine employees’ ability to navigate which attitudes and behaviors will be recognized in organizations and make decisions in attitudes holding and actions conducting. Past scholars found that empathy could induce altruism and cooperation (Batson and Moran, 1999), so this study involves altruism in the study of the influence of empathetic leadership on employees’ behaviors. Furthermore, based on social information processing theory, organizational climate can also influence how employees’ behaviors are formed (Salancik and Pfeffer, 1978). This study introduces the role of a caring ethical work climate in studying the influence of empathetic leadership because it can affect a broad range of decisions as general and pervasive characteristics provide support to all team members of organizations (Victor and Cullen, 1988). Finally, based on the social information processing theory, interactions among people and environments together shape employees’ behaviors. Hence, this study uses a configurational perspective to supplement the regression testing of the relationships among leadership, attitudes, climates, and behaviors.

Relying on regression analysis and fuzzy-set qualitative comparative analysis (fsQCA) based on survey data from a Chinese company, the authors of this work tend to contribute to the literature in several ways. First, though prior research has started focusing on the performance-related outcomes of empathic leadership (Kock et al., 2019), the study of empathetic leadership’s outcomes, particularly workplace behavior, has also been highlighted as a priority in further addressing (Ekiyor, 2019). Based on the social information processing theory, this study contributes to empathetic leadership literature by clarifying the relationship between empathetic leadership and employees’ organizational citizenship behavior. Second, we argue that previous research that associates empathetic leadership with performance does not comprehensively explain how empathic leadership influences workplace outcomes. As altruism can explain why prosocial-related behavior is taken (Pfattheicher et al., 2022), it can be used to link leadership and organizational citizenship behavior. Based on the social information processing theory, the study explains how altruistic attitude transfers to helping-others behaviors in organizations, which bridges the missing attitude-action link mentioned in past altruism literature (Bharthy and Sethi, 2020; Abdillah et al., 2022). Third, this research identifies the caring, ethical work climate as an essential factor in the information processing of empathetic leadership and organizational citizenship behavior. Unlike previous studies regarding ethical climate as a mediator (Aloustani et al., 2020), this study explores the moderating effect of the exogenous variable of a caring ethical climate to deepen the understanding of the boundaries of the influence of empathic leadership on organizational outcomes and enrich the ethical climate literature. Fourth, the complexity of the influence of leadership on organizational citizenship behavior still needs further investigation. This study contributes to the organizational citizenship behavior literature by identifying the configurational effects of empathetic leadership, altruistic attitude, and caring ethical climate in shaping high-level and low-level organizational citizenship behavior in the Chinese context, which also provides support for the applicability of the social information processing theory in the empathetic leadership literature and its basic assumptions. In sum, this study can reveal the role of empathetic leadership in achieving employees’ high-level organizational citizenship behavior.

## 2 Hypotheses development

### 2.1 Social information processing theory

Past literature has explored the feasibility of the application of the social information processing theory in studying the influences of leadership (Lu et al., 2019) and organizational climate (Liu et al., 2020) on employees. In the 1970s, social information processing theory was introduced to organizational contexts by Salancik and Pfeffer (1978), highlighting the importance of environmental influence and emotions in shaping individuals’ attitudes and behaviors (Lemerise and Arsenio, 2000). This theory conveys a few assumptions that guide researchers to view the world from a more complex perspective rather than a pure need-satisfaction perspective. The first assumption is that individuals do not live solely but interact with society and adapt their attitudes and behaviors to socially acceptable beliefs (Salancik and Pfeffer, 1978), which reflects that environmental influence can shape individuals’ behaviors. The second assumption is that individuals recognize and interpret phenomena and make emotional, cognitive, and behavioral reactions based on the information they receive (Salancik and Pfeffer, 1978). Based on these premises, by understanding individuals’ perceptions of cues conveyed in social interactions and environments, researchers may understand the generation and development of individuals’ attitudes and behaviors. According to the above assumptions, subordinates in AEC companies interact with colleagues under socially acceptable beliefs in workplaces. Furthermore, any changes in environments and interactions will influence their emotions and interpretations of the workplace, which influence their perceptions of organizations and behavioral responses. Hence, this study structures the hypotheses below based on this theory.

### 2.2 Empathetic leadership and employees’ altruistic attitude

Leaders play essential roles in interacting with employees in work life, and they can influence subordinates’ emotions and behaviors because they can profoundly affect subordinates’ resources and development (Hu and Shi, 2015). As subordinates regard leaders as representatives of organizations, they will navigate their ideas and status in organizations based on leaders’ responses to fit them into the organizations. According to the social information processing theory, subordinates will generate emotional, cognitive, and behavioral reactions after they proceed with cues received from the circumstances in which they live. In addition, Edmondson and Lei (2014) believe that employees need support, understanding, and empathy from organizations to satisfy their basic needs, which will improve employees’ mental health and increase their perceptions of organizations and performance in the workplace (Kock et al., 2019; Yrle et al., 2003). Hence, if subordinates receive positive, constructive, and supportive information from leaders, they will flourish because their psychological needs are satisfied, and they will ensure that their actions are aligned with social expectations.

Empathic leadership, as an emotional support leadership style, refers to the ability to recognize and understand the experiences of followers and, at the same time, provide emotional support to make others feel safe (Clark et al., 2019). According to the social information processing theory, subordinates will interpret their experiences and actions and make decisions about their later behaviors based on the information they receive from working life. As empathic leaders usually provide safety and emotional care to their followers, followers will maintain satisfying levels of basic needs and hold positive mental states (Wibowo and Paramita, 2022; Owens and Hekman, 2012). Furthermore, the supportive information sent by leaders may guide followers to better navigate their reactions because of the improvement of their sense of self-confirmation. Batson and Shaw (1991) defined altruism as a motivational state aiming to increase another’s welfare. When followers perceive empathic leadership as a process of sharing positive social information, they may generate altruistic attitudes in their workplace. First, person-care attitudes and support in empathetic leadership allow leaders to create emotional bonds with followers and establish a sense of in-group membership (Grant, 2013; Mayfield and Mayfield, 2009), which will motivate followers to help others (Yang et al., 2020). Second, an empathetic leadership process can inspire emotional contagion among followers (Barsade et al., 2018) and improve followers’ affect and positive feelings about the workplace, cultivating followers’ ability to express empathy in their daily work. Third, according to the empathy-altruism hypothesis (Seppälä et al., 2017; Khan et al., 2022), followers’ empathy will evoke altruistic attitudes, such as conferring benefits on others at their workplace. According to the above, this study hypothesizes:

*H1:* Empathetic leadership is positively related to employees’ altruistic attitudes.

### 2.3 Employees’ altruistic attitude and organizational citizenship behavior (OCB)

Organizational citizenship behavior (OCB) is traditionally defined as extra-role behavior that serves to advance organizations’ purposes. Five behaviors consist of OCB in Organ’s (1988) conceptualization: sportsmanship, altruism, conscientiousness, courtesy, and civic virtue. An altruistic attitude is the enduring tendency to think about the welfare and rights of other people, to feel concern for them, and to act in a way that benefits them (Van Emmerik et al., 2005). Although the dimension of altruism is included in organizational citizenship behavior (OCB), this dimension is different from the altruistic attitude discussed in this article. The altruistic attitude represents a relatively stable psychological and motivational tendency that is oriented towards the well-being of others, emphasizing the individual’s selfless care rather than the implementation of specific actions. In contrast, the altruism in organizational citizenship behavior (OCB) refers to the specific behaviors of helping others observed in the organizational context (Organ and Ryan, 1995). It should be noted that previous studies have pointed out that the helping behaviors in OCB may be driven by various motives, such as expectations of performance-related rewards or considerations of personal interests (Penner et al., 1997). Therefore, the altruism in OCB represents the observable behavioral outcomes, and these behaviors are not always derived from purely altruistic motives. Based on the above distinctions, this article regards the altruistic attitude as a psychological antecedent variable, which is used to explain under what circumstances employees are more likely to exhibit organizational citizenship behaviors, including altruistic behaviors, rather than considering it as a component of OCB itself.

Social information processing theory believes that people’s behaviors will be affected by their attitudes (Salancik and Pfeffer, 1978). When an employee generates altruistic attitudes after they interpret the information from the environment, he/she will choose to help others as a response to external information. Furthermore, when other people perceive, actively, politely, responsibly, and optimistically helping others without requiring re-wards, as an essential part of acceptable social norms, they will adapt their actions to social expectations by conducting similar actions, which will enhance the norms in organizations and inspire more extra-role behaviors in their workplaces. Since altruistic attitudes have been found to promote prosocial behaviors (Van Emmerik et al., 2005), employees in an organization who possess a strong altruistic attitude are not only more inclined to help others, but are also more likely to engage in other behaviors that are beneficial to the organization. Therefore, the strong relationship between altruistic attitudes and organizational citizenship behavior reflects its promoting effect as a generalized antecedent of multiple dimensions of OCB, rather than being a repetition of the concept. Thus, this study hypothesizes:

*H2:* Employees’ altruistic attitude is positively related to organizational citizenship behavior.

### 2.4 The mediating role of employees’ altruistic attitude

Based on social information processing theory, information in the workplace influences individuals’ attitudes, which in turn influences individual behaviors (Salancik and Pfeffer, 1978). When employees perceive their leaders as empathic ones, leaders’ behaviors and interpersonal communication can transmit powerful information and social clues to employees (Yam et al., 2018), which will influence their attitudes and behaviors. First, when empathetic leadership provides employees with instructional and emotional support during daily life and difficult occasions (Kock et al., 2019; Bani-Melhem et al., 2021; Mayfield and Mayfield, 2017), employees will feel that they are not alone because their personal needs will be satisfied. Regarding providing support to others as a socially accepted norm, employees will adjust their attitudes and adopt more altruistic attitudes. Second, immersed in supportive circumstances from leaders, employees will turn their altruistic attitudes into actions, which will motivate them to contribute to organizations. Furthermore, their altruistic actions will convey information to others and enhance the norms, which will improve the contagion effect and inspire other employees to conduct more extra-role behaviors in organizations to keep aligned with the norms of helping others. Accordingly, this study proposes that:

*H3*: Employees’ altruistic attitude mediates the relationship between empathetic leadership and organizational citizenship behavior.

Given the high pressure, ambiguity, and uncertainty characterizing the AEC industry, organizations increasingly depend on employees’ extra-role behaviors to maintain coordination (Liu et al., 2023). However, pressures from organizations due to the challenges they face can lead to AEC employees’ unethical and non-compliant behaviors and negative attitudes toward their roles (Saleem et al., 2022; Li and Ning, 2022; Yan et al., 2025; De Clercq and Belausteguigoitia, 2024). Under current global conditions marked by economic volatility and intensified performance demands, ethical work climates are proposed as normative cues to shape employees’ moral interpretations and behavioral priorities (Liu et al., 2022; Victor and Cullen, 1988).

According to Victor and Cullen (1988) and Martin and Cullen (2006), the most frequently observed behaviors in instrumental ethical climates involve self-serving and unethical conduct, as decision-making is dominated by personal gain and low perceived moral risk. Empirical studies consistently link such climates to opportunistic behaviors (Liu et al., 2022), and withdrawal from discretionary responsibilities (Zhou et al., 2024), particularly under performance pressure. Then, rules climates are most commonly associated with defensive noncompliance, characterized by strict adherence to formal procedures to avoid blame or punishment (Duh et al., 2010), which leads to low-level in extra-role or prosocial actions, especially in high-accountability project environments.

Within law and code climates, the most prevalent behaviors center on compliance with legal and professional standards (Jahanzeb and Raja, 2024), while discretionary or relational prosocial behaviors are often viewed as outside formal role boundaries. Consequently, helping behaviors beyond prescribed duties occur less frequently and remain narrowly defined. By comparison, independence climates lack a dominant behavioral pattern, as employees act according to personal moral standards, producing heterogeneous and situationally contingent outcomes rather than consistently enacted prosocial engagement.

Different from other ethical climates, caring ethical climates are among the few contexts in which ethical norms and discretionary prosocial behaviors are structurally aligned rather than inherently in tension (Leung, 2008). Caring for climates plausibly increases the likelihood that prosocial behaviors are enacted as morally appropriate responses, even under pressure. Accordingly, the achievement of harmony between the ethical climate and prosocial behaviors in organizations today should be understood as the outcome of interactions among ethical climate functioning as a critical contextual cue, other organizational conditions and employees’ individual cognitions. The next section therefore focuses on the role of caring ethical climate in our study.

### 2.5 The moderating role of a caring ethical climate

Based on the social information processing theory, individuals’ attitudes and behaviors are shaped by information from the circumstances in which they live. Subordinates’ attitudes and behaviors are not only influenced by leaders’ responses but also by their perceptions of the general circumstances of the organizations. Hence, it is necessary to investigate the boundary conditions in the relationship between leadership and subordinates’ attitudes and organizational citizenship behavior. In this study, a caring ethical work climate is regarded as a boundary condition. Ethical work climates reflect the perceptions and attitudes of employees toward ethical issues related to the enterprise (Victor and Cullen, 1988), which can effectively affect employees’ thinking styles, ethical judgment, and work behaviors in organizations (Teresi et al., 2019; Fu and Deshpande, 2014). Victor and Cullen (1988) summarized five types of ethical work climates, including caring, law and code, rules, instrumental, and independence. They emphasized that ethical climates are products of shared values and perceptions, and that they vary across organizations depending on contextual factors such as industry characteristics, national context, and organizational structure. Empirical studies have documented the presence of law and code ethical climates (Liu et al., 2022), caring ethical climates (Liu et al., 2022), rule ethical climates (Jha et al., 2017), independence ethical climates (Bulutlar et al., 2025), and instrumental ethical climates (Liu et al., 2022) in the AEC industry across different regions, but they are not evenly distributed. Given the project-oriented, interdependent and highly pressured nature of the AEC industries, organizations often face strong coordination needs and shared performance risks (Liu et al., 2022). Under such circumstances, a climate based on solidarity, such as caring ethical climate, is more likely to be actively emphasized and constructed as a normative framework because it highlights responsibility sharing and peer support. In contrast, climates that prioritize instrumental factors or highly individualistic elements may not be consistently emphasized in such environments because they may not fully align with the collaborative requirements inherent in complex project work. Among the dimensions of ethical climate, literature reveals that the caring dimension was most influential one in shaping employees’ ethical attitudes and behaviors (Fu and Deshpande, 2012). Thus, investigating how subordinates react to ethical dilemmas in workplaces is meaningful in understanding subordinates’ behaviors.

Based on the social information processing theory, in an organization with a high level of caring ethical climate, an employee will receive ethical information from the harmonious and ethical atmosphere constructed by leaders and colleagues’ caring actions in the organization, which motivates them to evaluate job characteristics to make corresponding behaviors to benefit the organization (Salancik and Pfeffer, 1978). If employees do not receive friendly information from the circumstances, they may perceive that their actions do not match the organizational norms, which constrains their altruistic attitudes and behaviors. Therefore, we formulate the following hypotheses:

*H4a*: A caring ethical climate moderates the relationship between altruistic attitude and organizational citizenship behavior such that this relationship becomes stronger when a caring ethical climate is high.

**FIGURE 1:**
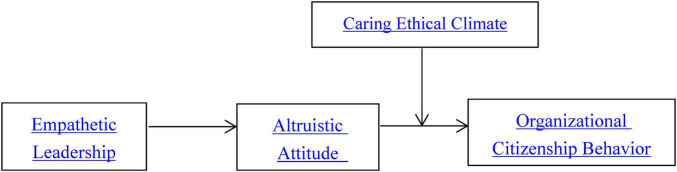
Research model

Based on the above hypotheses, the caring ethical climate may also moderate the indirect effect of leadership on employees’ behavior via attitudes. When an empathetic leader creates an emotional tie with employees by focusing on addressing employees’ needs, taking their feelings into account, and acting in a way that satisfies their needs and wants (Batson and Moran, 1999), employees will follow leaders’ actions and hold altruistic attitudes toward others. On this occasion, if they feel a high-level caring ethical climate, they will confirm their evaluations and altruistic attitudes and engage in further behaviors to benefit organizations. Therefore, we predict the following:

*H4b*: A caring ethical climate moderates the relationship between empathetic leadership and organizational citizenship behavior (via altruistic attitude), such that this relationship becomes stronger when a caring ethical climate is high. Based on the above part, Figure 1 shows the research model of this study.

## 3 Materials and methods

### 3.1 Sample and collection procedures

This study employed a three-wave time-lagged survey design. Survey data were collected from employees of a real estate company in North China. Participants were recruited using a non-probability convenience sampling approach. Specifically, employees who had direct work interactions with their supervisors were invited to join in the research. Given the possibility of sample selection error arising from the single-organization and non-probability sampling design, we hold cautious interpretations of the results regarding external generalizability.

With the help of the company’s HR manager, the first researcher gave a brief online introduction of this study to employees in the organization, explaining the purpose of this study and related ethical issues. 328 employees were invited to participate in this study. The data was obtained from a three-wave collection to control the risk of common method variance (CMV) (Podsakoff et al., 2003).

During the data collection period, at Time 1, the HR manager was invited to distribute paper-based surveys to employees on a voluntary basis. 328 employees voluntarily participated in the research and completed measures of leaders’ empathetic leadership, their altruistic attitude and demographic variables. A total of 319 employees completed the first survey. The response rate was 97.3%. To align responses across survey waves while preserving anonymity, the research team assigned anonymous identification codes to respondents in the first wave survey. One month later, the research team sent the second online survey link (supported by Wen Juan Xing, a famous Chinese survey assistance platform) to those employees filling out the first survey and asked them to report their perceptions of the organization’s caring ethical climate (Time 2). At this stage, 308 of them completed the second survey. Finally, 308 employees were asked to report their organizational citizenship behavior 1 month after the second survey via an online survey link (Time 3). After deleting incomplete survey data, the research team retained data from 296 samples for data analysis (Mean of age = 33.94, Standard Deviation of age = 6.44).

The participants did not receive any monetary compensation. However, in order to express gratitude and encourage participation, all respondents who completed all three rounds of the survey were eligible to enter a lottery. The prizes were three gift cards worth 100 yuan each. Additionally, we provided a summary report of the research results to the company’s human resources department for internal reference.

According to Holtom et al. (2022), this study’s response rates of surveys are at normal levels (the norm response rate level is 70% if managers distribute surveys, while the response rate should be 68% if researchers dis-tribute surveys). Of the participants, 49.3% were males, 50.7% were females. Regarding educational level, 12.2% of participants had completed junior college or below, 58.8% held an undergraduate degree, 27.7 % held a master’s degree, and 1.4 % held a doctoral degree.

Finally, Harman’s single-factor test shows that the first factor explains 27.5% of the variance (less than 50%), which reflects that common method bias is not a serious concern (Podsakoff et al., 2003).

### 3.2 Measures

As all participants were Chinese and not proficient in English, they were asked to respond to the Chinese version of the questionnaires. Following the back-translation procedure recommended by Brislin (1986), the authors translated the original version of the questionnaires into Chinese versions and proofread the Chinese versions with the help of two bilingual professors and two professionals in the AEC industry to ensure accurate and precise translation. Except for the control variables, all focused variables in this study were measured in the form of a Likert 5-point scale, with 1–5 ranging from “strongly disagree” to “strongly agree.”

Empathetic leadership: The five-item scale from Kock et al. (2019) was adopted, which assesses empathetic leadership as a one-dimensional construct. An example item is “My supervisor shows me encouragement for my work efforts” (Cronbach’s α = 0.92).

Altruistic attitude: A twenty-item scale developed by Philippe Rushton et al. (1981) was used to measure respondents’ altruistic attitudes as a one-dimensional construct. A sample item is “I have given directions to a stranger” (Cronbach’s α = 0.94).

Caring ethical climate: Victor and Cullen (1988) developed a scale to measure ethical work climate, including items designed to measure caring in organizations. Hence, this study adopted seven items of the caring aspect to measure respondents’ perception of caring climate as a one-dimensional construct. A sample item is “In this company, people look out for each other’s good” (Cronbach’s α = 0.86).

Organizational citizenship behavior: A 14-item scale developed by Williams and Anderson (1991) was used to measure respondents’ organizational citizenship behaviors in organizations. A sample item is “Helps others who have heavy workloads” (Cronbach’s α = 0.95). Although organizational citizenship behavior has been conceptualized as a two-dimensional construct comprising individual-directed and organization-directed behaviors, consistent with prior research focusing on higher-order constructs (Xu et al., 2021), the items were aggregated to form an overall OCB construct in this study.

Control variables: Past literature shows that people are differentiated in empathetic cognitions with different demographic backgrounds (Decety and Yoder, 2016), leading to various reactions to interpersonal relationships and organizational citizenship behavior (Dwivedi and Kaushik, 2015). Following the approach described by prior research (Van Emmerik et al., 2005), the authors controlled demographic variables (gender, educational level, marital status, and age) during statistical analysis. Except regarding age as a continuous variable, the authors set other control variables as categorical variables. Regarding gender, the authors used 0 to represent males and 1 to represent females. Subsequently, different educational levels are marked as: (1) high school degree, (2) college degree, (3) master’s degree, and (4) doctoral degree. Finally, people were grouped as the unmarried (0) and the married (1).

Validity and reliability: This study conducted a confirmatory factor analysis (CFA) to evaluate the construct validity with MPLUS 8.3. Table 1 shows that the four-factor model has a better model fit compared with other alternative models. The measurement model demonstrated acceptable absolute fit indices (RMSEA = 0.049 < 0.08, SRMR = 0.052 < 0.08) and satisfactory incremental fit indices (CFI = 0.909 > 0.9, TLI = 0.904 > 0.9), supporting the distinctiveness of the constructs (Medsker et al., 1994). Furthermore, this study assessed the convergent validity based on factor loadings, composite reliability (CR) and average variance extracted (AVE). Table 2 shows that all CR values exceeded the recommended threshold of 0.70 (Fornell and Larcker, 1981), and all factor loadings were statistically significant and above 0.40, which is considered acceptable in confirmatory measurement models (Lam, 2012; Mwansisya et al., 2021). Although the AVE values for two constructs were slightly below the suggested cutoff of 0.50, prior research indicates that convergent validity can still be considered adequate when CR values are high (Fornell and Larcker, 1981; Lam, 2012).

**TABLE 1:** Results of confirmatory factor analysis (CFA).

Models	*χ ^2^*	*df*	*Δx^2^*	RMSEA	SRMR	CFI	TLI
A+B +C+D	1693.542	983		0.049	0.052	0.909	0.904
A+B, C, D	2524.863	986	831.321	0.073	0.075	0.802	0.792
A+C, B, D	2443.340	986	749.798	0.071	0.082	0.812	0.803
A+D, B, C	2649.717	986	956.175	0.076	0.095	0.786	0.775
B+C, A, D	2450.319	986	756.777	0.071	0.082	0.811	0.802
B+D, A, C	3812.498	986	2118.956	0.098	0.132	0.636	0.618
C+D, A, B	2505.145	986	811.603	0.094	0.095	0.804	0.795

*N* = 296; ***p < 0.001; A, Empathetic leadership; B, Altruistic attitude; C, Caring ethical climates; D, OCB; RMSEA, Root mean square error of approximation; SRMR, Standardized root-mean-square residual; CFI, Comparative fit index; TLI, Tucker–Lewis’s index.

**TABLE 2:** Measurement validity and reliability.

Construct	Item	Loading	Cronbach’s α	AVE	CR
Empathetic leadership	EL1	0.846	0.92	0.69	0.92
	EL2	0.854			
	EL3	0.847			
	EL4	0.797			
	EL5	0.812			
Altruistic attitude	AL1	0.628	0.94	0.47	0.94
	AL2	0.702			
	AL3	0.735			
	AL4	0.734			
	AL5	0.522			
	AL6	0.612			
	AL7	0.780			
	AL8	0.641			
	AL9	0.741			
	AL10	0.634			
	AL11	0.576			
	AL12	0.642			
	AL13	0.651			
	AL14	0.622			
	AL15	0.585			
	AL16	0.749			
	AL17	0.614			
	AL18	0.654			
	AL19	0.741			
	AL20	0.662			
Caring ethical climate	CEC1	0.764	0.86	0.46	0.85
	CEC2	0.772			
	CEC3	0.811			
	CEC4	0.858			
	CEC5	0.467			
	CEC6	0.504			
	CEC7	0.427			
Organizational citizenship behavior	OCB1	0.774	0.95	0.55	0.94
	OCB2	0.804			
	OCB3	0.740			
	OCB4	0.688			
	OCB5	0.753			
	OCB6	0.793			
	OCB7	0.779			
	OCB8	0.785			
	OCB9	0.661			
	OCB10	0.719			
	OCB11	0.723			
	OCB12	0.647			
	OCB13	0.745			
	OCB14	0.778			

## 4 Results

### 4.1 Descriptive statistics and correlations

Table 3 shows the descriptive statistics (means and standard deviations) and the correlation analysis between the variables. Overall, the correlation patterns are consistent with the proposed hypotheses and provide preliminary support for subsequent hypothesis testing. Empathetic leadership was positively correlated with altruistic attitude (*r* = 0.41, *p* < 0.001), explaining approximately 17% of the variance in altruistic attitude (*r*^2^ = 0.17). This association was statistically robust, as indicated by the corresponding z-statistic (*z* = 7.46). Empathetic leadership was also positively associated with organizational citizenship behavior (*r* = 0.29, *p* < 0.001), accounting for approximately 8% of the variance (*r*^2^ = 0.08; *z* = 5.07). In addition, altruistic attitude showed a significant positive correlation with organizational citizenship behavior (*r* = 0.31, p < 0.001), explaining about 10% of the variance in organizational citizenship behavior (*r*^2^ = 0.10; *z* = 5.48). Caring ethical climate was positively correlated with both empathetic leadership (*r* = 0.21, *p* < 0.001; *r*^2^ = 0.04; *z* = 3.65) and altruistic attitude (*r* = 0.22, *p* < 0.001; *r*^2^ = 0.05; *z* = 3.82), although its correlation with organizational citizenship behavior was not statistically significant.

**TABLE 3:** Descriptive statistics and correlations for variables (*N* = 296).

Variables	Mean	SD	1	2	3	4	5	6
1. Gender	0.51	0.50	0.02	0.31***	0.07	0.41*** (0.17) *z* = 7.46	0.22*** (0.05) *z* = 3.82	−0.07 (0.01) *z* = 1.20
2. Age	33.94	6.44						
3. Marital	1.82	0.38	0.01					
4. Empathetic leadership	3.08	1.22	-0.01	0.05				
5. Altruistic attitude	3.00	0.87	0.03	0.00	0.00			
6. Caring ethical climates	3.28	1.01	0.10	0.07	−0.07	0.21*** (0.04) *z* = 3.65		
7. OCB	3.29	0.90	−0.06	0.05	0.03	0.29*** (0.08) *z* = 5.07	0.31*** (0.10) *z* = 5.48	

***p < 0.001. The numbers in parentheses are values of r^2^; the numbers below are the *z*-values.

### 4.2 Hypotheses testing

**FIGURE 2:**
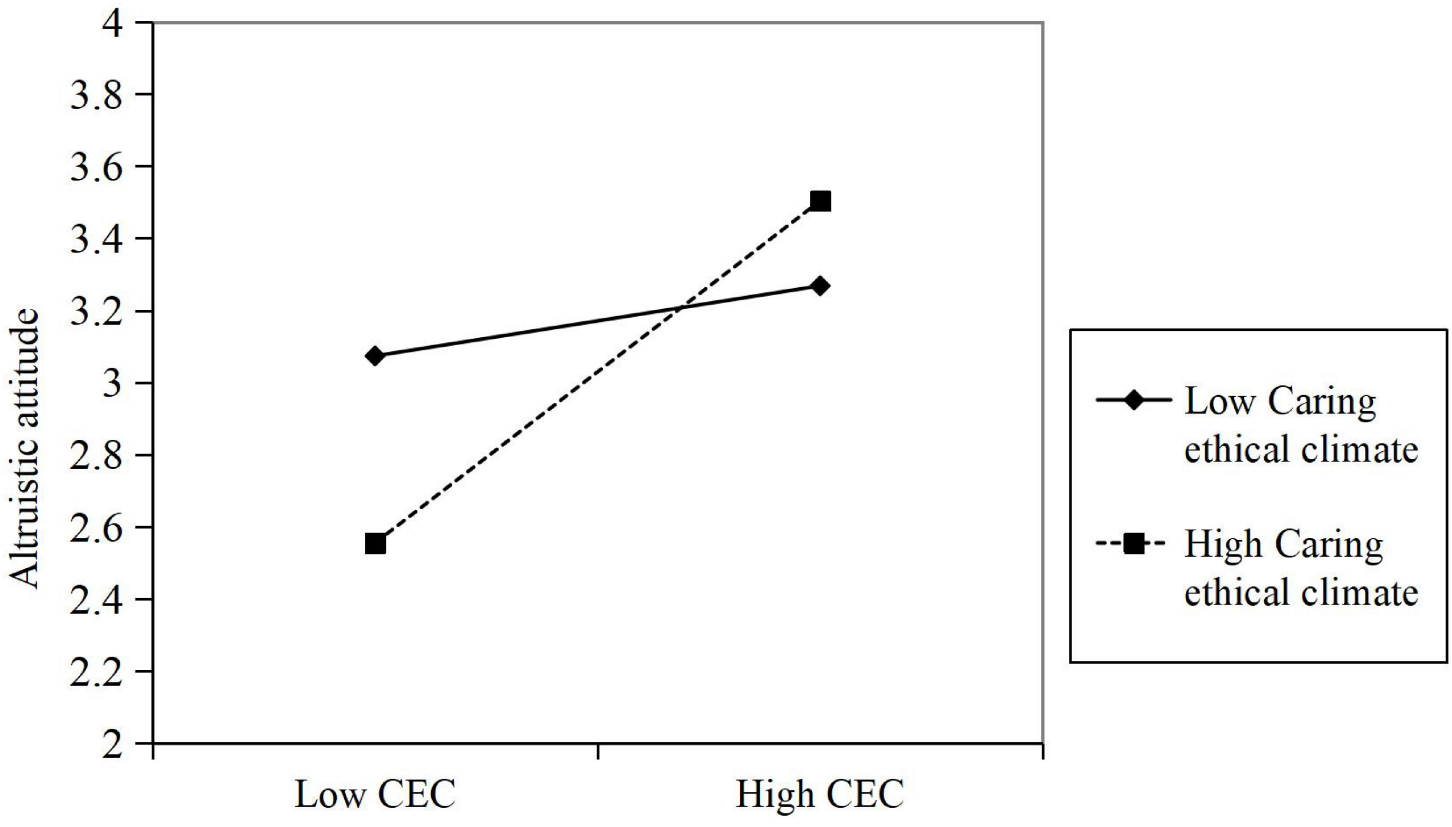
The moderating effect of a caring ethical climate.

H1 and H2 were tested by multiple linear regression analyses. Table 4 presents the results from SPSS 22. As shown in M1 of Table 4, empathetic leadership is positively and significantly related to altruistic attitude (β = 0.41, *p* < 0.001), which supports hypothesis 1. As shown in M3 of Table 4, altruistic attitude is positively and significantly related to employees’ organizational citizenship behaviors (β = 0.31, *p* < 0.001), which supports hypothesis 2. Subsequently, H3 was tested using mediation model 4 in the SPSS PROCESS macro based on 2,000 bootstrapped repeated samples (Hayes, 2014). The results are shown in Table 5. Supporting H3, the results reveal the positive and indirect effect of empathetic leadership on organizational citizenship behavior through altruistic attitude (β = 0.14, boot SE = 0.05, 95% CI = [0.05, 0.23]). Then, H4a and H4b were tested using model 14 in the SPSS PROCESS macro based on 2,000 bootstrapped repeated samples. As shown in M5 of Table 4, the interaction between altruistic attitude and caring ethical climate is positively correlated with organizational citizenship behavior (β = 0.21, *p* < 0.001), reflecting that the moderating effect of caring ethical climate on the relationship between altruistic attitude and organizational citizenship behavior is significant. Hence, H4a is supported. Finally, H4b posits the moderated mediation effect of the caring ethical climate. Supporting H4b, the analysis reports that the moderated mediation effect is significant (moderated mediation index: β = 0.05, boot SE = 0.02, 95% CI = [0.08, 0.10]). Specifically, the mediating effect of altruistic attitude is statistically significant under the conditions of a high-level caring ethical cli-mate (β = 0.13, boot SE = 0.03, 95% CI = [0.08, 0.20]) but is not significant at a low-level caring ethical climate (β = 0.03, boot SE = 0.03, 95% CI = [-0.03, 0.08]) (Table 5). Figure 2 shows the moderating effect of a caring ethical climate on the relationship between empathetic leadership and organizational behavior.

**TABLE 4:** Unstandardized coefficient and standard error of moderated mediation path analysis.

Independent variables	Altruistic attitude			OCB		
	M1	M2	M3	M4	M5	M6
Gender	0.03	−0.05	−0.07	−0.06	−0.06	−0.05
Age	−0.01	0.04	0.05	0.04	0.05	0.05
Marital	−0.02	−0.01	0.01	−0.00	−0.01	−0.02
Empathetic leadership	0.41***	0.29***		0.20**		0.18**
Altruistic attitude			0.31***	0.23***	0.32***	0.25***
Caring ethical climates					−0.08	−0.11
Altruistic attitude **×** caring ethical climates					0.21***	0.17**
*ΔR* ^2^	0.17	0.08	0.10	0.05	0.04	0.03
*ΔF*	58.18	26.77	31.53	15.08	14.02	9.62

** indicates *p* < 0.01, *** indicates *p* < 0.001.

**TABLE 5:** Results for the mediation and moderated mediation model.

Level of caring ethical climates	Indirect effect	SE	95%CI
**Simple mediation effect**			
	0.14	0.05	[0.05, 0.23]
**Moderated mediation effect**			
High caring ethical climates	0.13	0.03	[0.08, 0.20]
Low caring ethical climates	0.03	0.03	[−0.03, 0.08]

### 4.3 Supplemented Fuzzy-set qualitative comparative analysis

As fsQCA can reveal the asymmetric relationships among variables, it allows researchers to understand the nonlinear and combined effects of variables in shaping consequences. Hence, this study uses fsQCA 4.1 to run fsQCA as a supplemented analysis technique to investigate the interactive effects of leadership, climate, and individuals in shaping organizational behaviors. The fsQCA is conducted in three steps: calibration, necessity analysis, and sufficient conditions analysis (Fainshmidt et al., 2020).

Calibration: In the first calibration step, this study followed past literature and used 5, 50, and 95% as thresholds to compute the original data of variables except for gender (0 = male or 1 = female) and marital status (0 = unmarried or 1 = married) into values ranging between 0 and 1 (Fainshmidt et al., 2020). The values computed based on three thresholds for variables are exhibited in Table 6. Furthermore, this study converts the sample whose values were calibrated as 0.5–0.499 to prevent software from neglecting the observation due to the ambiguity of calibration (Williams and Anderson, 1991).

**TABLE 6:** Calibration.

Variable	95%	50%	5%
EL	4.8	2.9	1.4
AL	4.25	2.85	1.75
OCB	4.429	3.429	1.643
CEC	4.571	3.429	1.55
AGE	45	33	26
EDU	3	2	1

Necessity analysis. In the necessity analysis step, this study set 0.9 as the cutoff point for the consistency scores of all conditions (Ragin, 2008). Table 7 shows that none of the conditions is an always necessary condition for organizational citizenship behavior because all the values of consistency are less than 0.9.

**TABLE 7:** Necessity analysis.

Condition	OCB		∼ OCB	
	Consistency	Coverage	Consistency	Coverage
Gender	0.493212	0.515847	0.522032	0.484153
∼Gender	0.506788	0.544569	0.477968	0.455432
Marital status	0.834987	0.536869	0.812301	0.463131
∼Marital status	0.165014	0.497846	0.187700	0.502154
EL	0.687992	0.714044	0.531535	0.489183
∼EL	0.507821	0.550049	0.689288	0.662048
AL	0.700996	0.713822	0.541635	0.489079
∼AL	0.498260	0.550738	0.683070	0.669504
CEC	0.621300	0.634691	0.651054	0.589761
∼CEC	0.598417	0.659163	0.596725	0.582857
EDU	0.710296	0.652807	0.708913	0.577745
∼EDU	0.540559	0.676816	0.573982	0.637271
AGE	0.607073	0.680014	0.572372	0.568530
∼AGE	0.614811	0.618517	0.677852	0.604704

Then, this study conducted a sufficiency analysis based on the initial truth tables, setting 0.8 as the raw consistency threshold (Papadopoulos et al., 2013), 0.75 as the PRI consistency threshold (Li et al., 2022), and 0.75 as the cut-point of the consistency to check the degree of informativeness of the models (Li et al., 2022). Following Ragin’s (2008) recommendation and past literature (Woodside, 2013), this study reports the intermediate solutions. Table 8 shows a sufficient situation for causal reasoning because six interpretation paths for high-level OCB cover approximately 88% of the cases (overall consistency of the model = 0.88 > 0.8). The coverage of the model (0.4) reflected that it explained 40% of the factors leading to high-level OCB. Subsequently, the consistency values of the six configurations were 0.910, 0.911, 0.895, 0.916, 0.909, and 0.935, respectively. It showed that the conditions were sufficient to explain OCB. Similarly, the consistency values of configurations and the total model for low-level OCB were higher than 0.8, suggesting the reliability of the configurations. Furthermore, these configurations could explain 36.7% of the cases.

**TABLE 8:** Configurational analysis.

Solution	High-level OCB						Low-level OCB			
	1	2	3	4	5	6	1	2	3	4
EL	•	•	•	•	•	•	⊗	⊗	⊗	•
AL		•	•	•	•	•	⊗	⊗	⊗	⊗
CEC	•	•	•		•	⊗	•	•	•	⊗
Age		⊗	⊗	•	•	⊗	⊗	⊗	•	⊗
Gender	⊗	•	⊗	⊗		•		•		•
Marital status	•	⊗	•	•	•	•			•	⊗
Education	•			•	•	•	⊗			•
Consist.	0.909529	0.91069	0.895221	0.916089	0.90887	0.934559	0.935031	0.94565	0.921171	0.970075
Raw cover	0.178144	0.0428979	0.121446	0.148294	0.258101	0.0910291	0.219321	0.147582	0.251157	0.0279623
Unique cover.	0.028231	0.0428979	0.00894296	0.0196896	0.0597638	0.0212959	0.044064	0.0213492	0.0990615	0.0122919
Solution Consist.	0.879708						0.920335			
Solution cover	0.400468						0.367047			

“•” represents the presence of a condition, “⊗” represents the absence of a condition, and a blank space represents either presence or absence.

Regarding the high-level OCB, there are six configurational paths. Configuration 1 is ∼gender*marital status*EL*CEC* education. The consistency of it is 0.910, explaining 17.8% of samples’ high-level organizational citizenship behavior. This configuration shows that subordinates have stronger intentions to conduct organizational citizenship behaviors when they are married and highly educated males who are led by high-level empathetic leadership and have been working in a high-level caring climate in organizations.

Configuration 2 is gender*∼marital status*EL*AL*CEC* ∼age. The consistency of it is 0.911, explaining 4.3% of samples’ high-level organizational citizenship behavior. This configuration shows that subordinates have stronger intentions to conduct organizational citizenship behaviors when they are unmarried and young females with altruistic attitudes and have been led by high-level empathetic leadership and have been working in a high-level caring climate in organizations.

Configuration 3 is ∼gender*marital status*EL*AL*CEC* ∼age. The consistency of it is 0.895, explaining 12.1% of samples’ high-level organizational citizenship behavior. This configuration shows that subordinates have stronger intentions to conduct organizational citizenship behaviors when married, and young males with altruistic attitudes have been led by high-level empathetic leadership and have been working in a high-level caring climate in organizations.

Configuration 4 is ∼gender*marital status*EL*AL* education*age. The consistency of it is 0.916, explaining 14.8% of samples’ high-level organizational citizenship behavior. This configuration shows that subordinates have stronger intentions to conduct organizational citizenship behaviors when they are married, well-educated, and old males with altruistic attitudes and have been led by high-level empathetic leadership in organizations.

Configuration 5 is marital status*EL*AL*CEC* education*age. The consistency of it is 0.909, explaining 25.8% of samples’ high-level organizational citizenship behavior. This configuration shows that subordinates have stronger intentions to conduct organizational citizenship behaviors when they are married, old, well-educated, and altruistic and have been led by high-level empathetic leadership and when they have been working in a high-level caring climate in organizations.

Configuration 6 is gender*marital status*EL*AL*∼CEC* education*∼age. The consistency of it is 0.935, explaining 9.1% of samples’ high-level organizational citizenship behavior. This configuration shows that subordinates have stronger intentions to conduct organizational citizenship behaviors when they are married, well-educated, and young females with altruistic attitudes and have been led by high-level empathetic leadership and when they have been working in a low-level caring climate in organizations.

Four paths exist for achieving low-level organizational citizenship behaviors (Table 8). Configurations 1, 2, and 3 show that when empathetic leadership and altruistic attitude are maintained at low levels, organizational citizenship behaviors will less appear even if employees work in a caring climate. Furthermore, Configuration 4 shows that when altruistic attitudes and a caring climate are maintained at low levels, organizational citizenship behaviors will be less likely to appear, even if leaders conduct empathetic leadership practices to inspire employees. This study also tested the robustness by changing the case consistency threshold (i.e., increasing the threshold from 0.8 to 0.85). Table 9 shows that the conclusions are robust because the configurations do not have visible change after the case consistency threshold is changed.

**TABLE 9:** Robust check (case consistency threshold is 0.85): configurational analysis.

Solution	High-level OCB						Low-level OCB			
	1	2	3	4	5	6	1	2	3	4
EL	•	•	•	•	•	•	⊗	⊗	⊗	•
AL		•	•	•	•	•	⊗	⊗	⊗	⊗
CEC	•	•	•		•	⊗	•	•	•	⊗
Age		⊗	⊗	•	•	⊗	⊗	⊗	•	⊗
Gender	⊗	•	⊗	⊗		•		•		•
Marital status	•	⊗	•	•	•	•			•	⊗
Education	•			•	•	•	⊗			•
Consist.	0.909529	0.91069	0.895221	0.916089	0.90887	0.934559	0.935031	0.94565	0.921171	0.970075
Raw cover.	0.178144	0.0428979	0.121446	0.148294	0.258101	0.0910291	0.219321	0.147582	0.251157	0.0279623
Unique cover.	0.028231	0.0428979	0.00894296	0.0196896	0.0597638	0.0212959	0.044064	0.0213492	0.0990615	0.0122919
Solution consist.	0.879708						0.920335			
Solution cover.	0.400468						0.367047			

“•” represents the presence of a condition, “⊗” represents the absence of a condition, and a blank space represents either presence or absence.

## 5 Discussion

### 5.1 Findings

To understand the roles of empathy and ethical climate in inspiring extra-role behavior within the AEC industry, which urgently requires extra-role behaviors due to project-based work structures, high task interdependence, strict deadlines, and an uncertain environment, we examined how empathetic leadership and organizational circumstances jointly shape employees’ organizational citizenship behaviors. The regression analysis reveals that a caring ethical climate positively moderates the relationship between empathetic leadership and organizational citizenship behavior via employees’ altruistic attitudes. This finding suggests that empathetic leadership is particularly effective in the AEC context when it is embedded in a supportive moral environment, which is aligned with McCauley et al.’s (2024) work (2024) who revealed that empathy can stimulate prosocial behavior through selflessness, and also resonates with Liu and Chan (2017) who argue that organizational climate can interact with leadership to inspire employees’ behaviors in the AEC industry.

Regarding the supplemented fsQCA analysis, although the results show that none of these factors can solely inspire employees’ organizational citizenship behaviors, empathetic leadership remains essential in enabling high-level organizational citizenship behaviors, highlighting the configurational and context-sensitive nature of such behaviors in the project-based AEC industry. Several typical configurations leading to high-level organizational citizenship behaviors were identified. First, when leaders demonstrate high levels of empathetic leadership and cultivate a caring ethical climate, married and highly educated males, unmarried and young females with altruistic attitudes, married and young males with altruistic attitudes, or married, older, and well-educated altruistic employees (regardless of gender) are likely to engage in high-level organizational citizenship behaviors. Subsequently, when leaders demonstrate high-level empathetic leadership practices alone, subordinates who are married, well-educated, and older males with altruistic attitudes tend to display high-level organizational citizenship behaviors. Finally, when leaders demonstrate high-level empathetic leadership while operating within a low-level caring ethical climate, married, well-educated, and young females with altruistic attitudes are more likely to engage in high-level organizational citizenship behaviors. In contrast, the fsQCA results indicate that the absence of altruistic attitudes constitutes a primary pathway leading to low-level organizational citizenship behaviors, underscoring the importance of employees’ internal moral orientations.

The findings of this study provide important insights into how organizational citizenship behavior can be understood and sustained in the AEC industry. The integrative perspective directly addresses the research objective by explaining why extra-role behaviors are both essential and fragile in the AEC industry, where formal control mechanisms are often insufficient and informal cooperation becomes a critical organizational resource.

### 5.2 Theoretical implications

Our findings contribute to literature in several aspects. First, this study conducted an empirical test to confirm the importance of empathic leadership in the AEC industry’s leader-employee relationship. Although the links between empathetic leadership and job performance and resilience have been tested by scholars, more studies of empathetic leadership outcomes are still needed. This study verifies organizational citizenship behavior as a specific positive outcome of empathetic leadership, which contributes to the empathetic leadership aspect of empathy literature.

Second, this study extends the findings of Murat Eminoğlu and Elçi (2023) on the positive relationship between organizational empathy and helping behavior, revealing that the leaders’ empathetic interactions can inspire their altruistic attitudes and organizational citizenship behaviors. This mechanism can verify the possibility of emotion-based triggers rather than traditional exchange assumptions in leader-member interactions, providing an example of studying leader-member interactions from an emotional perspective. Furthermore, this mechanism explains how altruistic attitudes transfer to helping other behaviors in organizations, which bridges the missing attitude-action link mentioned in past altruism literature (Bharthy and Sethi, 2020; Abdillah et al., 2022).

Third, this study responds to the scholars’ call for rich, caring, ethical climate research on the relationship between leadership style and employee behavior (Sembiring et al., 2020). Past literature usually regards the ethical work climate as a mediator (Aloustani et al., 2020) in the lead-er-outcome relationship. Referring to the social information processing theory, the results show that the indirect effect between empathetic leadership and organizational citizenship behavior was stronger for those in a higher caring ethical climate, which not only enriches the empathetic leadership literature but also provides a reference to future research in studying the role of ethical work climate in other leaderships’ influences.

Fourth, although past scholars have started studying empathy in organizations, few of them regard the influence of empathy as a complex process but regard empathy as a trait or specific behavior. This study verifies the applicability of the social information processing theory in empathetic leadership research, which provides another angle from which to view the influence of empathy in leadership in organizations and proposes the potential of theories established based on the assumption of social construction.

Fifth, the supplemented qualitative comparative analysis verifies the complexity of the initiation of high-level employees’ behaviors in an empathetic leadership context. It reveals the conditions as non-solo necessary conditions but combined effects of leadership, climate, and attitudes in shaping actions, and lacking altruism as the main reason for low-level organizational citizenship behavior, which makes not only a methodological contribution to empathetic leadership literature but also proposes meaningful paths for the emergence of low-level and high-level organizational citizenship behavior in Chinese organizational context research.

### 5.3 Practical implications

The findings of this study are meaningful to practitioners. This study supports Jian’s (2022) statement that empathetic leadership benefits managers and organizations in the Chinese AEC industry. The study revealed that providing leader support for followers can increase followers’ altruistic attitudes when their psychological and safety needs are satisfied. Hence, enterprises may establish empathetic leadership training to indirectly inspire employees’ altruistic attitudes. Furthermore, enterprises may encourage frontline managers to take care of employees’ situations and provide support. For instance, if a leader notices that a follower is experiencing problems sleeping because of a new infant in the house, he/she can express understanding of the follower’s situation and provide the employee with a quiet workspace to take a short nap during break times. These humane practices will make employees recover soon and maintain health and may inspire employees’ helping attitudes and actions toward colleagues. Subsequently, the study found that a caring ethical climate can amplify the influence of empathetic leadership on employees’ actions because their altruistic attitudes will be further confirmed by supporting information from the circumstances. Hence, enterprises may organize team-building activities and provide room for employees so that they may feel support from the organizations and conduct further organization-interested practices. Finally, the supplemented fsQCA analysis reveals that the appearance of high-level organizational citizenship behaviors in organizations is complex and shaped by leaders, environmental factors, and individuals. Additionally, organizational citizenship behaviors can be inspired by different configurations of influential factors. Therefore, managers may flexibly conduct specific actions to inspire employees with different demographic variables to raise organizational citizenship behaviors, such as providing educational background progress support or organizing fast dating activities.

## 6 Conclusion and Limitations

### 6.1 Conclusion

Acknowledging the challenges in addressing stress and improving performance in the AEC industry, we examine the influencing mechanism of empathic leadership on organizational citizenship behavior based on survey data from a Chinese real estate company. Based on social information processing theory, this study verifies the influence of empathetic leadership on organizational citizenship behavior via an altruistic attitude in a caring ethical work climate context. Furthermore, the fsQCA analysis identifies several paths shaping high-level organizational citizenship behavior. The findings can contribute to empathetic leadership, altruistic attitude, ethical work climate and organizational citizenship behavior literature, providing evidence for the emotion-driven cognition mechanism in linking leadership and employees’ organizational citizenship behavior in the AEC industry.

### 6.2 Limitations and future research

Although this study contributes to literature in several ways, it also has a few limitations. First, all variables were reported by employees due to access restrictions for supervisors in the organization, which may raise concerns about CMV. To alleviate the CMV concerns, we utilize a time-lagged design to form a three-wave survey and apply a statistical technique to ensure the influence of CMV is limited. Second, although the measurement for empathetic leadership in this study used to be applied to assessing empathy, the applicability of the established scale for evaluating empathetic leadership has been verified in this study. To comprehensively understand the empathetic leadership phenomenon, future researchers should create a measure specifically targeted to capture the full spectrum of empathetic leadership in the Chinese context because Eastern Asians may understand empathy differently from Westerners who develop the existing empathy scale. Third, our data supports the importance of the caring, ethical climate in understanding the effect of empathetic leadership on organizational citizenship behavior. However, it is also possible that the other boundary conditions may be used to explain the above relationship. Thus, we encourage future studies to contribute to the empathetic leadership literature by examining the moderating impact of other variables. Fourth, although the configurational effects of variables on organizational citizenship behavior have been investigated in this study, the reasons for the initiation of different kinds of high-level organizational citizenship behavior may be diversified. Hence, further research should explore the configurational effects of various elements in shaping more specific types of employees’ in-role and extra-role behaviors, which will contribute to empathetic leadership literature and social information processing theory development.

## Data Availability

The raw data supporting the conclusions of this article are available from the corresponding author upon reasonable request.
